# Diatom Cooccurrence Shows Less Segregation than Predicted from Niche Modeling

**DOI:** 10.1371/journal.pone.0154581

**Published:** 2016-04-29

**Authors:** Marius Bottin, Janne Soininen, Didier Alard, Juliette Rosebery

**Affiliations:** 1 Irstea Bordeaux, UR EABX, 50 avenue de Verdun, 33612, Cestas cedex, France; 2 Univ. Bordeaux, INRA, BIOGECO, 33615, Pessac, France; 3 Department of Geosciences and Geography, University of Helsinki, PO Box 64, Helsinki, FIN-00014, Finland; Universidade Federal de Goiás, BRAZIL

## Abstract

Species cooccurrence patterns give significant insights into the processes shaping communities. While biotic interactions have been widely studied using cooccurrence analyses in animals and larger plants, studies about cooccurrences among micro-organisms are still relatively rare. We examined stream diatom cooccurrences in France through a national database of samples. In order to test the relative influence of environmental, biotic and spatial constraints on species’ incidence distribution, cooccurrence and nestedness patterns of real communities were compared with the patterns generated from a set of standard and environmentally constrained null models. Real communities showed a higher level of segregation than the most conservative standard null models, but a general aggregation of cooccurrences when compared to environmentally constrained null models. We did not find any evidence of limiting similarity between cooccurring species. Aggregations of species cooccurrences were associated with the high levels of nestedness. Altogether, these results suggested that biotic interactions were not structuring cooccurrences of diatom species at our study scale. Instead, the patterns were more likely to be related with colonization patterns, mass effect, and local temporal dynamics of diatom biofilms. We further highlight that the association of standard and environmentally constrained null models may give realistic insight into the cooccurrence patterns of microbial communities.

## Introduction

The forces underlying local community structure and the relationship with larger scale processes are central topics in community ecology. Assembly rules refer to the way local communities are built from species pools [1]. Depending on the ecological processes considered, they can be separated into three groups [2]: (i) dispersal assembly rules (i.e., dispersal filters the species that enter a local site from the species pool), (ii) abiotic assembly rules (environmental preferences select the species, i.e., niche assembly) and (iii) biotic assembly rules (constraints related to the competition, facilitation or any other biotic interaction). On a large scale, assembly rules and ecological processes are regularly inferred from the measurements and tests of patterns they are expected to produce [2]. Here we focus on the processes that drive species’ incidences and on cooccurrence patterns at a regional scale. Apart from environmental preferences of species studied through niche modeling, biotic interactions and dispersal processes are classically considered the most important drivers of species’ incidences.

Competition is expected to constrain local occurrences of species through competitive exclusion. A major assumption in community ecology theory is that competition is greater between similar species than between species with dissimilar traits [3,4]. This process results in a pattern of segregation between species that is particularly strong between similar species. Such a pattern is accentuated by niche complementarity, i.e. communities with a high functional diversity are favored because of more efficient resource use. Moreover, facilitation is likely to concern functionally dissimilar species, which would result in accentuating the segregation between similar species. Therefore, one can expect (i) general segregation between species due to competitive exclusion, and (ii) “limiting similarity” between coexisting species in the resulting communities.

Dispersal processes and assembly rules are often studied through the prism of metacommunities. Depending on the relative importance of environmental filters and dispersal processes, ecologists use four main theoretical types of metacommunity organization [5,6]: (i) patch dynamics, (ii) species sorting, (iii) mass effect and (iv) neutrality. These four different paradigms imply different levels of order in the species’ incidence data at the regional scale, typically measured by the level of nestedness [7,8]. Nestedness corresponds to the degree to which relatively species-poor assemblages are proper subsets of species-rich assemblages, i.e. rare species are not typically found in species-poor assemblages [9]. Moreover, this pattern arises from structured gradients of richness and commonness of species [8,10] and its measurement assesses the importance of large scale patterns and the associated processes driving the local occurrence distribution. Nestedness represents a concentration of occurrences in rich sites, i.e. occurrences in a nested matrix are “aggregated” in the richest sites. Therefore, we expect a negative relationship between nestedness and segregation patterns. Nevertheless Ulrich and Gotelli [11] showed that the opposite relationship between nestedness and aggregation of cooccurrences could arise in cases where simulated communities were obtained under a lognormal drawing model and consequently that the patterns were mathematically independent.

Both segregation and nestedness are extremely sensitive to the differences of diversity between localities and taxonomic groups. The assessment of their significance requires the use of appropriate null models. Such null models must meet one main requirement: to include all the processes acting on real communities except the one you wish to test for [12]. Recently, the shortcomings of null models have been pointed out, and avenues for their improvement have been suggested [2,13]. One of the major shortcomings of traditional null models is that they overlook of species' niches and their relationships with the local environment [2,13–15]. Since the diverse environmental preferences of species can lead to clear differences in their occurrences, accounting explicitly for species responses to environment can lead to a better understanding of the role of other processes in shaping communities, such as dispersal or biotic interactions. Some studies have accounted for species’ environmental niches by assessing the cooccurrences between species while checking for their habitat preferences [16] or by constructing predictive models of occurrences from environmental variables [17]. Moreover, Peres-Neto et al. [18] developed environmentally constrained null models, and various authors have used this approach for various specific goals [19–21].

Most of the studies using null models to infer assembly rules investigated vascular plant or animal communities. Studies of assembly processes in micro-organisms are relatively rare, although more and more datasets are becoming available [22]. Benthic diatoms are unicellular algae and play an important role in the biogeochemical cycles of rivers, and they are particularly diverse. They are widely used in biomonitoring programmes as they are known to respond particularly fast and predictably to environmental changes. Because their ecological preferences are relatively well known, benthic diatoms also provide an interesting model for disentangling the effects of niche responses from other ecological processes on species distribution. Diatom’s siliceous cell walls allow a morphological description of the species, similar to those of macroscopic organisms, which also makes them good model organisms. Moreover, diatom assemblages, like other structured microbial communities in biofilms, exhibit succession patterns comparable to those of higher plants [23] with a growth in height and increasing intensity in biotic interactions during transitions from one succession stage to another, and as biofilm ages [24].

Our main aim here was to unravel species cooccurrence patterns of diatoms. Our method compares the real structure of diatom occurrences to those of pseudo-communities inferred from standard and environmentally constrained null models. By using different null models simultaneously, our goal is to offer a framework in order to disentangle the main processes that shape cooccurrence patterns. We applied two types of null models to create sets of pseudo-communities. These pseudo-communities were compared with real communities using (i) standard null models consisting of a randomization procedure of real communities, and (ii) environmentally constrained null models using probabilities of presences of each species based on predictions by environmental variables.

Specifically, we tested the following hypotheses:

1. Due to inherent structuring of communities and ecological constraints such as biotic and/or dispersal assembly rules, segregation patterns of real assemblages should depart from both purely stochastic models and niche modeling predictions.
2. Due to competition and niche complementarity, cooccurrence of functionally similar species should be less likely than cooccurrence of dissimilar species.
3. Nestedness should be associated with a general aggregation of species occurrences.

## Materials and Methods

### Data

**Fig 1:**
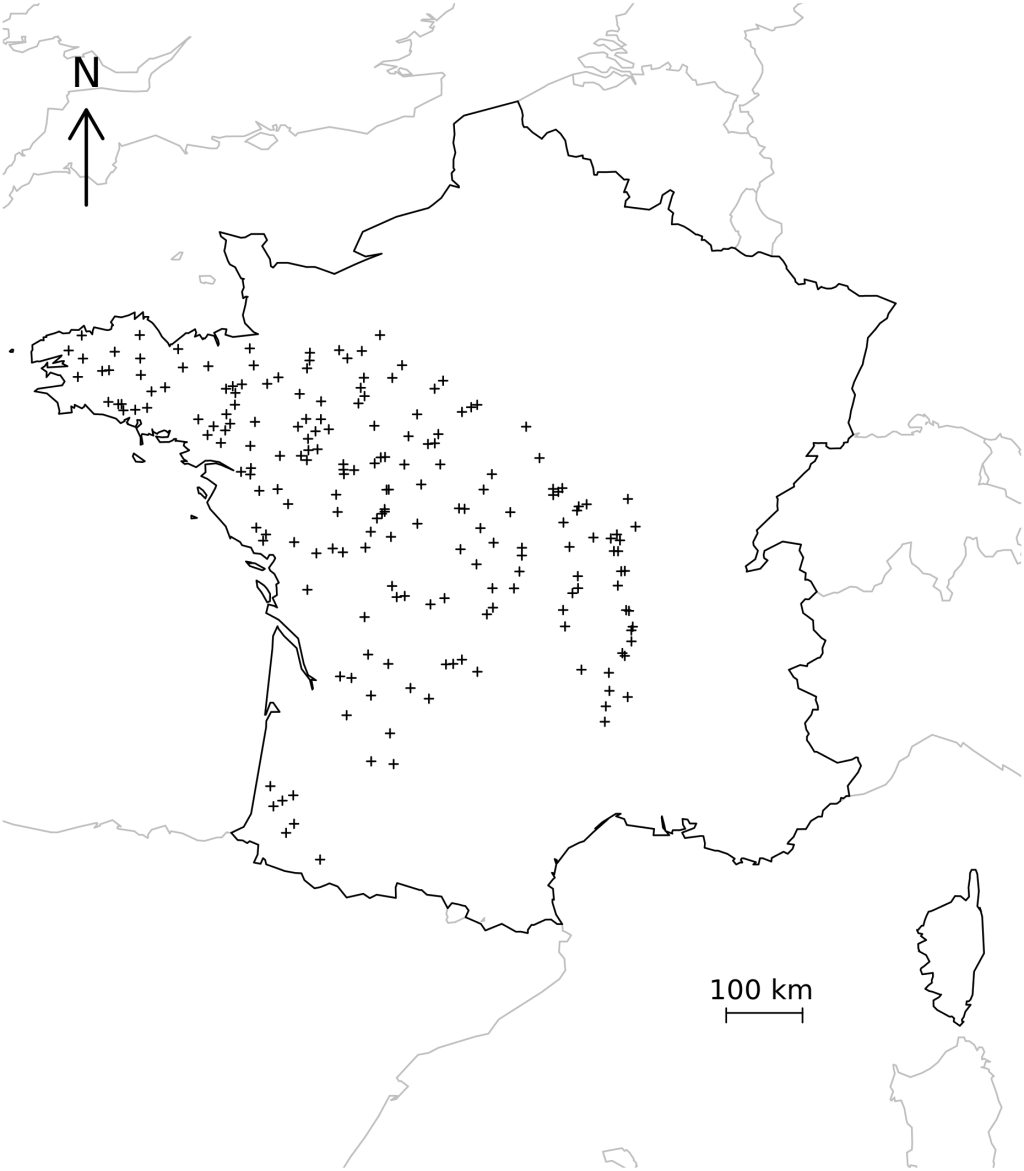
Location of sampling sites in France.

Our database consists of 196 samples collected in French rivers and streams (Fig 1) in 1998 by the Cemagref (France) and the Centre de Recherche Gabriel Lippman (Luxembourg). In order to avoid excessive dependence between samples collected at closely located sites, sampling sites less than 2 km from each other were removed from the data. All samples were collected from stones and during the low flow period (June to September) and analyzed by only two experts according to a standardized method (NF T90-354[25]), in order to limit variability due to the season and to local factors like sampling substrate. Diatom taxa were identified at 1000× magnification (Leitz DMRD light microscope) (400 valves per slide were counted to determine the presence of the species) according to Krammer and Lange-Bertalot [26] by examining permanent slides of cleaned diatom frustules, digested in boiling H_2_O_2_ (30%) and HCl (35%) and mounted in a high refractive index medium (Naphrax, Northern Biological Supplies Ltd, UK; RI = 1.74). Forms, varieties and subspecies were lumped together in order to obtain a biologically coherent dataset and to minimize differences in identification between analysts. Species present in less than five samples were excluded from the data. The biological data matrix was composed of [196 samples x 264 species].

#### Environmental data

Geographic information (altitude (m), distance from the source (m, after determining the most remote upstream point of the river, i.e. the source, in a comprehensive spatial river database, the distance was calculated following the river sections), slope (%)) and physical-chemical data (pH, conductivity at 20°C (μS/cm), dissolved oxygen (mg/l), total inorganic nitrogen (mg/l), phosphate (mg/l), calcium (mg/l), hydrogen carbonate (mg/l), temperature (°C), suspended solids (mg/l), ammonium (mg/l), nitrate (mg/l), nitrite (mg/l), alkalinity (meq/l)) were collected for each site. Physical-chemical data, depicting local environment and the important environmental gradients for diatoms, were provided by the French “Agences de l'Eau” (“Water agencies” in charge of water policy in the six main French basin). Average values across data from the diatom sampling month and the values from the month before the sampling were used for every local variable in data analyses. The data table consisting of geographical information and physical-chemical data will be further referred to as “environmental data”.

#### Species grouping

In order to disentangle the overall patterns of species cooccurrences between functional or phylogenetic groups, we analyzed the patterns for different functional (guilds) and phylogenetic (genera) groups of species.

Diatom guilds were proposed by Passy [27] and they consist of three classes of species, depending on the growth forms and positions of species in the biofilm. The three classes are (i) “high profile” species, which are present in the overstory of the biofilm, (ii) “low profile” species, which are limited to the understory of the biofilm, and (iii) “motile” species with the ability to move in the biofilm. The guilds were mainly attributed at the genus resolution, except for species known to have a particular growth form in their genera, following Passy [27]. Additional information was included from various sources [23,28–30]). Among the listed species, 58 were considered as “low profile”, 66 as “high profile” and 120 as “motile” species. We did not have sufficient information to attribute a guild for the remaining 20 species, which were grouped as “unknown” guild. Results for the “unknown” guild are available in the supporting information (S3 Table).

### Data analyses

#### Distance-based redundancy analysis

In order to describe the variation of species' occurrences across sites and their relationships with the environment, we used a constrained ordination method called “distance-based redundancy analysis” [31,32]. Asymmetrical constrained analyses allow the linear relationships between the species' main gradients and environmental predictors to be described. Whereas standard redundancy analysis consists of a constrained version of Principal Component analysis, dbRDA is a constrained version of Principal Coordinates analysis, based on a distance matrix calculated on the community data. Here we used the Jaccard index of dissimilarity to describe the main species’ incidence gradients. A stepwise variable selection procedure was used to select only the most relevant environmental variables in the model based on the Akaike’s Information criterion. An ANOVA-like permutation test allowed us to assess the significance of environmental constraints. dbRDA were computed with the *capscale* function of package *vegan* [33] in R.

#### Standard null models

In order to verify the non-randomness of the cooccurrence patterns, we used null models to create sets of random matrices. Several standard null models exist depending on the constraints in terms of fixed sums of rows/columns [34]. One of the most widely used null models consists of the random redistribution of the species' presences to the different sites of the biological matrix, each species being drawn independently. We applied this procedure to create 1000 complete randomized matrices, and we will further refer to this null model as “NullModFE”.

Null models with fixed row and column sums are more difficult to compute, but have shown interesting statistical properties [34–36], because they preserve the among-sample differences in species richness and among-species differences in occurrence frequencies. Among these algorithms, the “trial swap” algorithm [37] has been considered the most suitable because resulting matrices are equiprobable samples of all possible matrices. This type of null model is here referred to as "NullModFF".

Miklós and Podani [37] further suggested that the number of trials in each step should be set such that the expected number of swaps equals twice the number of presences in the matrix. However, our preliminary results did not support this option (some matrices were similar to the preceding ones) and we thus used numbers of swaps tenfold the number of presences in the matrix. Before computation, matrices were initialized with twice the number of swaps during actual computing. We used this algorithm to sequentially obtain 1000 random matrices. This algorithm was computed with an adapted version of the C-program provided in the appendixes of Miklós and Podani [37]. This code was then used in R. The R and C sources are available from the authors upon request.

#### Niche Modeling

In order to describe the fraction of the cooccurrence pattern explainable by environmental differences between sampling units, we predicted species presences based on environmental data. Predictions were estimated in a leave-one-out procedure (presence in each site is predicted with models learning on all the data except the one to be predicted), using logistic regressions [38] and random forest models [39]. We obtained presence scores (between 0 and 1) for each species and each sampling unit.

Multiple logistic regression describes the relationship between a combination of predictors (here environmental data) and a binary response variable [38], here presence or absence of the focal species. An automatic stepwise procedure based on Akaike Information Criterion was used to select relevant variables for each species with *stepAIC* and *glm* functions of the *MASS* package in R [40]. Then predictive models using these variables were applied for each site by models constructed on every site except the focal one, and to predict the score using environmental data of the focal site. Responses from all the models were arranged in a “pseudo-probability” matrix of the same size as the real presence/absence matrix.

We then used random forest as a supervised classification method to predict scores (pseudo-probabilities) of presence and absence of species. Random forest models [39] belong to the decision tree family. Decision trees allow data partition in order to assign objects (here: vectors of environmental data) into classes (here: presence or absence of species). The random forest constructs a large number of decision trees, each with a randomly selected amount of data. Output results of these random forests are “votes”: each decision tree predicts a class for the input vector. The prediction of a class by one tree is considered as a vote for this class. Random forest models were computed with the *randomForest* package in R [41]. We used 500 trees for each forest. Due to the robustness of random forest, we did not use any early selection of variables for these models, but directly the leave-one-out procedure. We kept proportions of presence votes (“pseudo-probabilities”) as output of models.

#### Predicted pseudo-community matrices

Predictions led to matrices, similar to the real presence/absence matrices, but with values ranging from 0 to 1 (instead of only 0/1 values). With these scores (pseudo-probabilities), we applied sampling scheme procedures accounting for these pseudo-probabilities in order to reconstruct presence matrices. These procedures correspond to environmentally constrained null models, because the only factor they account for is the relationships between each species presence and the environmental data in our database.

In the first procedure, we drew as many presences in each site as in the real matrix (i.e., we kept local species richness fixed) depending on species’ pseudo-probabilities without replacement. This was repeated 1000 times for each site in order to produce 1000 pseudo-community presence matrices. For random forest and logistic regressions these procedures were called “RandForPF” and “LogitPF”, respectively.

The second procedure was less constrained and more driven by the pseudo-probabilities themselves. It consisted of drawing scores that were equally distributed between 0 and 1 to create matrices of the same dimensions as the pseudo-probability matrices. Pseudo-probabilities higher than equally distributed scores were considered as presences in a pseudo-community matrix. This was repeated 1000 times to obtain 1000 pseudo-community matrices. For random forest and logistic regressions these procedures were called “RandForPP” and “LogitPP”, respectively.

Table 1 gives a summary of the constraints on pseudo-community richnesses and commonness of the species.

**Table 1:** Summary of the pseudo-community models.

	Random	Logistic regression	Random Forest	Fixed richness	Fixed number of occurrences
NullModFF	X			X	X
NullModFE	X				X
LogitPF		X		X	
LogitPP		X			
RandForPF			X	X	
RandForPP			X		

Each model was used to obtain 1000 pseudo-community matrices.

#### Cooccurrence indices

Checkerboard units index consists of numbering species pairs forming perfect checkerboards ($\begin{array}{ll} \text{0} & \text{1}\\ \text{1} & \text{0} \end{array}$ or $\begin{array}{ll} \text{1} & \text{0}\\ \text{0} & \text{1} \end{array}$) in the overall matrix (considering all possible pairs of species and sampling units). It measures the importance of segregative patterns between species. Considering two species, searching for perfect checkerboard patterns is equivalent to calculating:

$$CU_{ij}=\sum \left(S_{i}-q\right)\left(S_{j}-q\right)$$

[42] where S_i_ and S_j_ are the numbers of samples where the species *i* and *j* are present, respectively, and *q* is the number of samples where both species are present. The sum of CU_ij_ considering all species pairs gives the general index for a biological matrix, but it is also possible to separate the calculation between different groups of species. In the different communities and pseudo-communities, we calculated checkerboard units between species from the same group (e.g. motile-motile) and between species from different groups (e.g. motile-high profile). In order to disentangle differences of global patterns between real communities and pseudo-communities from the different models, an index of over-estimation (or under-estimation) of checkerboard units in pseudo-communities was calculated for each pair of species’ groups as follows:

$$F=\frac{CU_{mod}}{CU_{real}}$$

where CU_real_ and CU_mod_ are the number of checkerboard units in the communities and pseudo-communities, respectively.

C-score is the average number of checkerboard units by species [43] and is calculated as follows:

$$C=\frac{\sum CU_{ij}}{n(n-\text{1})}$$

where n is the number of species in the matrix.

Micro-organisms are known to be efficiently dispersed at very large scales [44]. However, recent studies have shown that spatial patterns could also emerge at regional scales [45]. Therefore, the national scale in France (ca. 644 000 km^2^) appears to be an appropriate scale for examining diatom metacommunity patterns such as nestedness. The NODF index [46] has been proved to be consistent with the original concept of nestedness. NODF is based on the twin properties of standardized differences in presence–absence matrix row and column fills and paired overlap (i.e. the overlap of presences in two adjacent columns) resulting from a nested ordered sequence of nestedness. It was calculated in our data by using the “nestednodf” function of the package “vegan” [33] in R, and the values of NODF were compared with the general C-scores of the different community and pseudo-community matrices.

## Results

### Distance-based redundancy analysis

**Fig 2:**
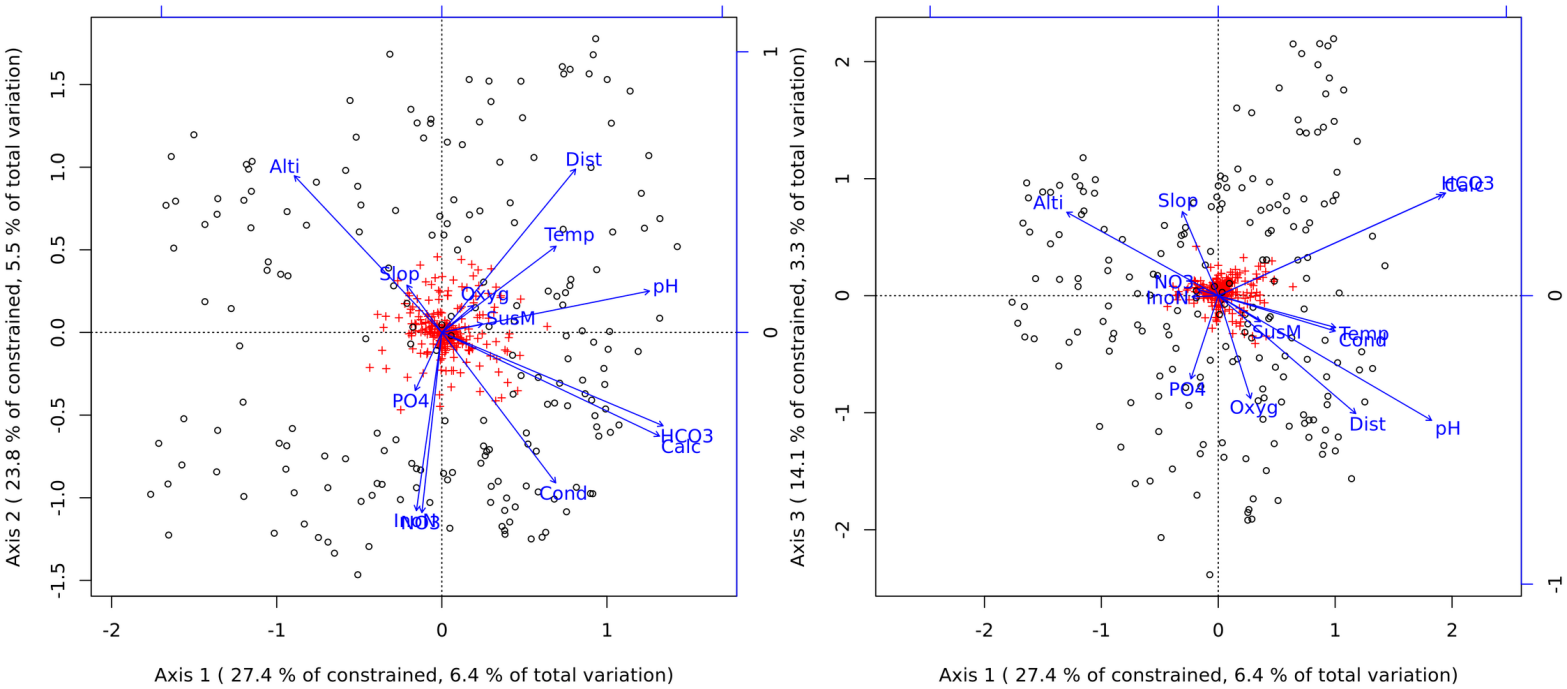
Triplot of the db-RDA. (Alti = Altitude, Dist = Distance from the source, Slop = Slope, Oxyg = concentration of dissolved oxygen, InoN = total inorganic nitrogen, PO4 = phosphate concentration, Calc = calcium concentration, HCO3 = hydrogen carbonate concentration, Temp = temperature, SusM = suspended solids concentration, NO3 = nitrate concentration)

The constrained part of the db-RDA includes 23% of the total inertia. The three first constrained axes include 65% of the constrained inertia, and 15% of the total inertia. The early selection procedure dropped three variables from the most comprehensive model: alkalinity (strongly correlated with hydrogen carbonates; R^2^>0.99), total inorganic nitrogen (strongly correlated with nitrates; R^2^ = 0.98) and nitrites. Nine constrained axes (out of 13 with positive eigenvalues) are found significant (Table 2). The most important variables in the model are the altitude and the distance from source, both of which were highly correlated with the first and second axes (Table 3 and Fig 2). The first axis also describes a gradient of alkalinity, whereas the second axis is strongly influenced by nitrates. The third axis describes a complex gradient including some remaining information about distance from source and pH, but is more specifically correlated with phosphates and dissolved oxygen (positively), and slope (negatively.). This axis seems to discriminate the nutrient rich oxygenated sites from lowland sites.

**Table 2:** Importance and significance of the constrained axes with positive eigenvalues in the distance based redundancy analysis (dbRDA) model.

	Sum of squares	Pseudo F
Constrained Axis 1	3.49	15.14 ***
Constrained Axis 2	3.03	13.16 ***
Constrained Axis 3	1.8	7.80 ***
Constrained Axis 4	1.08	4.69 ***
Constrained Axis 5	0.67	2.92 ***
Constrained Axis 6	0.55	2.36 ***
Constrained Axis 7	0.46	2.01 ***
Constrained Axis 8	0.39	1.71 **
Constrained Axis 9	0.32	1.39 *
Constrained Axis 10	0.28	1.2 (ns)
Constrained Axis 11	0.24	1.05 (ns)
Constrained Axis 12	0.22	0.97 (ns)
Constrained Axis 13	0.18	0.77 (ns)
Residuals	41.97	

Significance was assessed with a permutation test (1000 permutations) on the Pseudo-F. ns stands for non-significant,

“.” for P-value< = 0.1,

“*” for P< = 0.05,

“**” for P< = 0.01

“***” for P< = 0.001.

**Table 3:** Importance and significance of the variables in the db-RDA model.

	Sum.of.squares	Pseudo F
Distance from source	2.36	10.23 ***
Altitude	2.30	9.97 ***
Calcium	1.61	7.00 ***
pH	1.37	5.92 ***
Temperature	1.06	4.58 ***
Conductivity	0.65	2.81 ***
Nitrate	0.63	2.73 ***
Phosphate	0.59	2.54 ***
Dissolved oxygen	0.50	2.17 ***
Total inorganic nitrogen	0.48	2.09 **
Hydrogen carbonates	0.46	1.98 **
Slope	0.42	1.83 **
Suspended matter	0.31	1.34 .
Residuals	41.97	

Only the variables kept in the early stepwise selection procedure are shown. Significance was assessed with a permutation test (1000 permutations) on the Pseudo-F.

^“.”^ stands for P-value< = 0.1,

“*” for P< = 0.05,

“**” for P< = 0.01

“***” for P< = 0.001.

### C-scores

**Fig 3:**
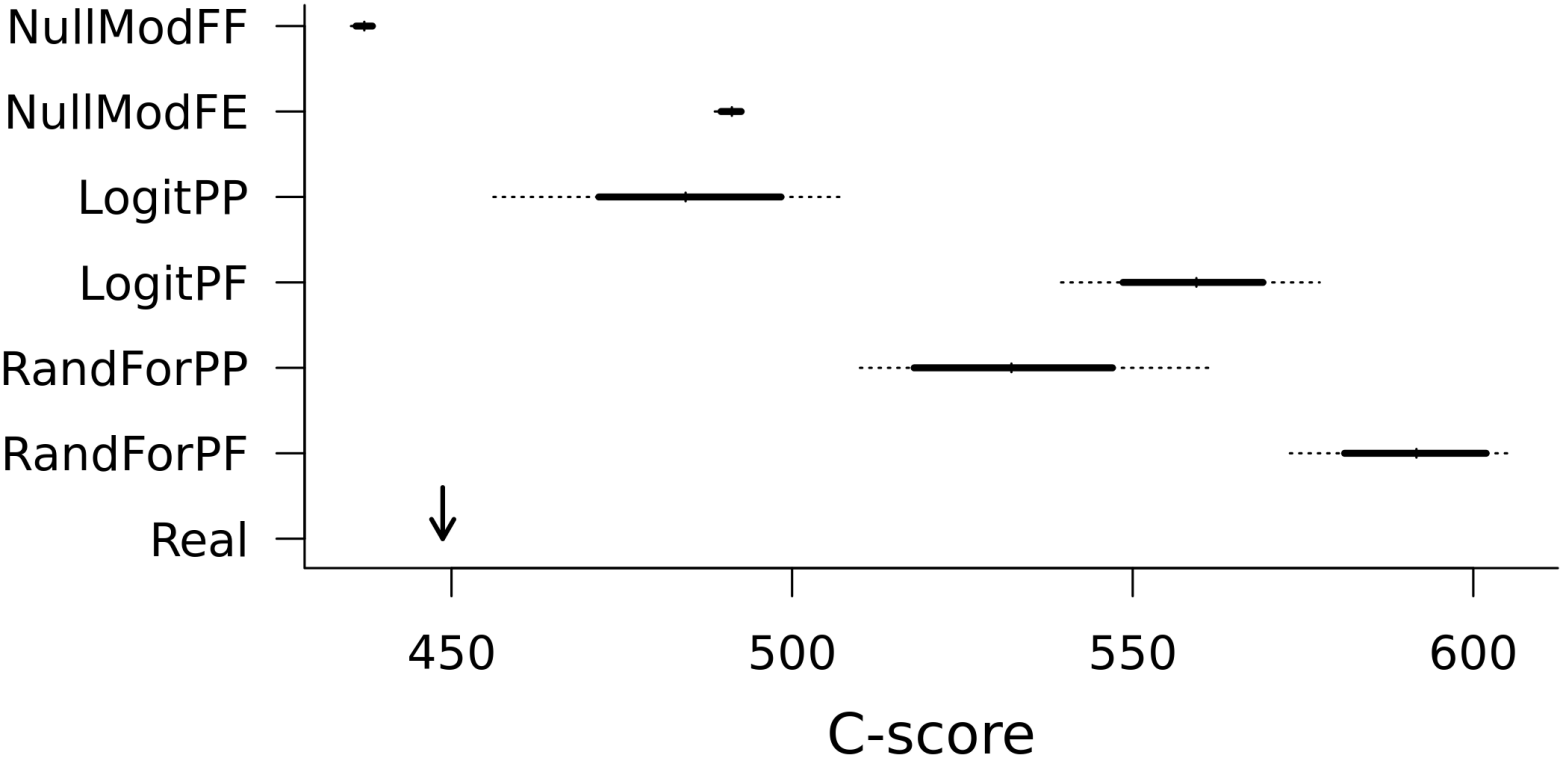
C-scores of pseudo-community matrices, in comparison to the C-score of the real matrix, in the different datasets. Bold segments represent 95% of the distributions. Perpendicular marks represent the median of distributions. The arrow indicates the value of the real community matrix. The models with names beginning by “NullMod” correspond to standard null models, those with names beginning by “Logit” correspond to environmentally constrained null models based on logistic regression predictions and those beginning by “RandFor” correspond to constrained null models based on Random Forest predictions. At the end of the name “FE” stands for occurrences Fixed and Equiprobable site richnesses, “FF” stands for occurrences and richnesses Fixed, “PF” stands for proportional occurrences (depending on pseudo-probabilities) and fixed richnesses, and “PP” stands for proportional occurrences and richnesses.

None of the pseudo-matrices obtained by the different standard or environmental null procedures produced C-scores equivalent to those of the real matrix (Fig 3).

All environmentally-constrained null models resulted in more segregative cooccurrence patterns than the real community matrix (Fig 3). NullModFE produced matrices with more segregative patterns than the real matrix as well. Finally, the NullModFF procedure led to a more aggregative pattern than the real matrix and the other models. The real matrix was located between the two standard null models concerning the aggregation patterns. The closest null distribution to the real value of the C-score was the one using NullModFF (standard null model with fixed raw and column sums). It is also interesting to note that the strong constraints in terms of commonness of the species (and richness for NullModFF) led to rather narrow distributions of the predicted C-scores, whereas the use of environmentally constrained null models allowed more flexibility in terms of commonness of the predicted species (result not shown) and wider distributions of the C-scores).

Constraining richness of samples in the environmental models led to more segregative patterns (LogitFP and RandForFP) and in models using logistic regressions it led to more aggregative patterns than those generated using Random Forest models. Only LogitFP pseudo-communities showed lower C-scores than NullModFE among the environment-accounting models.

### Genera and guilds

The averaged f-values presented in Tables 4 and 5 were characterized by very low standard errors of the means (always lower than 104). They were always significantly different from the general *f*-values (concerning all species without considering genera and guilds) presented in the tables.

**Table 4:** Average F-values for checkerboard units in the different models depending on species’ guilds.

	NullModFF	NullModFE	LogitPP	LogitPF	RandForPP	RandForPF
Total	**0.97**	**1.09**	**1.08**	**1.25**	**1.19**	**1.32**
Low-High	0.93	1.04	1.06	1.19	1.14	1.26
Low-Motile	0.94	1.06	1.05	1.21	1.14	1.28
High-Motile	0.98	1.10	1.08	1.23	1.19	1.30
Low-Low	0.94	1.06	1.08	1.23	1.14	1.30
High-High	0.99	1.11	1.09	1.23	1.20	1.30
Motile-Motile	1.03	1.16	1.12	1.31	1.24	1.39

The values shown are the averaged f values for the 1000 pseudo-communities. If values are higher than 1, the model overestimates the checkerboard units. In contrast, values below 1 indicate that model underestimates the number of checkerboard units. Comparison between the separate values and the corresponding total value reveals the particular distribution of segregation between species' guilds in the matrices resulting from models. For model names, see Table 1.

**Table 5:** Average F-values for checkerboard units in the different models depending on species’ genera.

	NullModFF	NullModFE	LogitPP	LogitPF	RandForPP	RandForPF
Total	**0.97**	**1.09**	**1.08**	**1.25**	**1.19**	**1.32**
Between	0.97	1.09	1.08	1.24	1.18	1.31
Within	1.07	1.20	1.13	1.33	1.27	1.42

“Within” corresponds to the sum of checkerboard units of species from the same genus, “between” concerns species pairs from different genera. Values shown are the averaged F-values for the 1000 pseudo-communities. If values are higher than 1, the model overestimates the checkerboard units. Values below 1 indicate that the model underestimates the number of checkerboard units. Comparison between the separated values and the corresponding total value reveals the relative importance of checkerboard estimations in the matrices resulting from models. For model names, see Table 1.

Segregation between pairs of species from the motile guild was always over-estimated in the pseudo-communities (Table 4), when compared to the real communities (F-values superior to 1), but also when compared to the average total F-value obtained by the models. The second and third highest F-values in most of the models concern pairs of species from the “high” guild and pairs including one “motile” and one “high” species respectively (these segregations tend to be overestimated as well in the models). In contrast, the models tend to overestimate segregation less (or underestimate it for NullModFF) for pairs including one or two low profile species.

In all models and datasets, pseudo-communities were characterized by a greater over-estimation of segregations between species of the same genus than between species from different genera (Table 5).

### Relationship between cooccurrence patterns and nestedness

**Fig 4:**
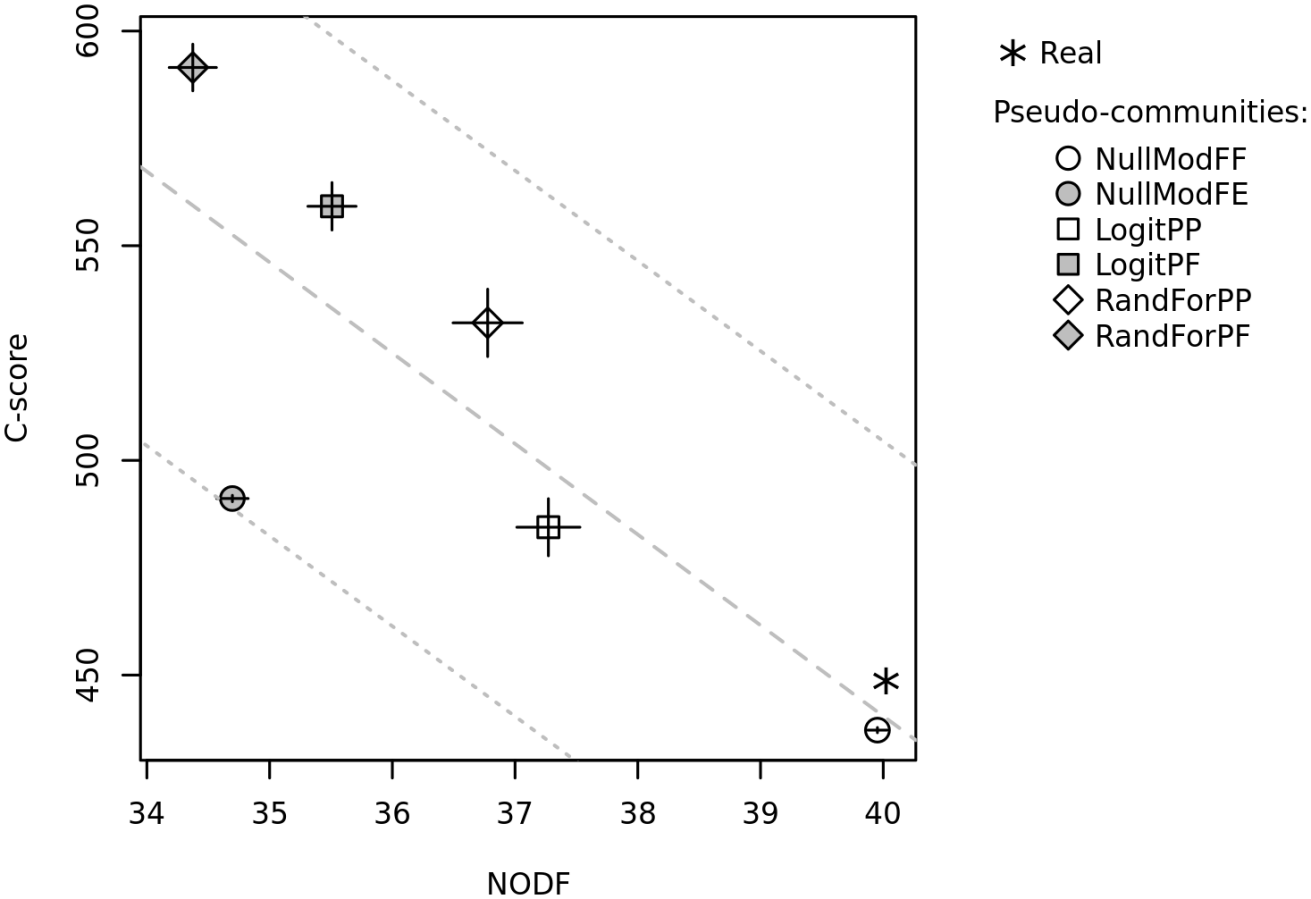
Relationship between C-scores and NODF nestedness indices of real and pseudo-communities. Lines crossing the symbols represent standard deviations of the pseudo-community indices. The dashed grey lines represent linear models fitted on the 6000 pseudo-communities. The dotted grey lines represent the predictive confidence interval of these models. For model names, see Table 1.

The NODF of the real matrix was higher than those calculated on the pseudo-communities except for NullModFF (Fig 4). Nestedness can thus be considered significantly higher than expected by environmental species preferences, but equivalent to that expected by chance when constrained richness and sum of occurrences are applied.

The relationship between NODF and C-score are shown in Fig 4. It was strongly negative in pseudo-communities, i.e. strong segregative patterns were associated with low nestedness (*P*<0.001). The real community value of the C-score is within the predictive confidence interval and the predicted C-score (439.98) is very close to the real one (448.71).

## Discussion

### More aggregation than expected from niche modeling

Supporting our first hypothesis, our results pointed out that real cooccurrence patterns departed from both random and environmentally predicted structure. These results support the hypothesis of assembly rules inherent to communities, neither random nor environmentally predictable, that drive segregation patterns between species, instead of a simple additive response of the different species. Real communities showed much lower segregation between species than the environmentally predicted pseudo-communities. This result is not consistent with the expected structure of communities in the presence of strong biotic relationships. However, our data and methods might not have been entirely suitable to infer biotic relationships at this scale.

First, our sampling protocol that pooled subsamples from several stones may have hidden possible interactive processes between species as these are likely to take place at very small scales in diatom communities. Azovsky [47] argued that micro-organisms perceive their environment at a finer grain than that considered here and cooccurrence in pooled samples may not imply real local coexistence. The same issue of scale has been documented for fish by Mouchet et al. [48] who argued that the sampling grain they used was larger than the scale at which competition actually takes place in fish communities. Second, it can also be argued that none of our models totally discard all biotic processes, resulting in a narcissus effect bias in the test of biotic interactions. Competition may be already integrated in the predicted species response to the environment because the data used actually reflect “species' realised niche” instead of the “fundamental niche”. Indeed, competition might have strongly affected overall occurrences of species, richness of sites or the environmental preferences of species ("Ghost of the competition past" [49,50]). While the effect of biotic interactions cannot be ruled out at all temporal and spatial scales, our results suggest that these interactions are not responsible for segregation patterns between species at the extent and grain of our study.

### No limiting similarity: due to temporal dynamics?

Our results did not support a “limiting similarity” assembly rule, as could be expected for strong biotic relationships in our second hypothesis. Instead, we found a general pattern of aggregation in real communities, particularly strong for functionally close species. In all null models, checkerboard units were generally more overestimated for pairs of species from the same genus or the same guild than for pairs of species from different genera or guilds. Many authors have failed to find evidence of limiting similarity patterns earlier [2,51,52]), despite the extensive theoretical literature advocating for communities composed of species functionally more different than expected by chance [1,53,54]). Cases of communities with greater cooccurrences between related species are classically attributed to the effects of environmental filters [48,52] (but see [21]). Missing environmental variables in the models might then explain our pattern, but adding variables similar to those used here would create even more segregation between species and make pseudo-communities depart even more from real communities in terms of C-scores.

Niche modeling particularly overestimated segregation when pertaining to motile species, known to develop at the end of successions [29,55]. The least over-estimated segregations were those involving low-profile species, and to a lesser extent high-profile species, respectively first and second to develop in the temporal succession of diatom biofilms. These results tend to show that the particular over-estimation of segregation between similar species is related to the temporal dynamics of diatom biofilms. The successional patterns in river diatom biofilms are characterized by a passive accumulation of species, referred to as “neutral coexistence” by Passy and Larsson [56] or as “passive tolerance” by McCormick and Stevenson [57]. New species develop in the biofilms neither inhibiting nor facilitating species which developed in previous phases. Thus, the cooccurrence patterns of the early-developing species are more easily linked to their environmental niches because of their rapid development. A clear relationship between pioneer low-profile species and their environmental niche explains the relatively lower over-estimations of segregation patterns involving these species. On the other hand, late developing species are more likely to be found in mature biofilms. We find them more aggregated than one would expect from their measured environmental preferences, which explains the strong over-estimation of their segregations in the models. Even though the patterns observed should be experimentally tested, these results stress the importance of considering temporal succession patterns in the distribution of diatom species.

### Nestedness: a link between aggregation and large-scale patterns

As expected in our third hypothesis, we found a strong relationship between local segregation of species' occurrences and the general nestedness pattern at the study scale. The strong linear relationship between the Nodf and C-score index constructed on the pseudo-communities allowed us to accurately predict the C-score of the real matrix. This result allows us to draw a potential link between the processes driving local segregation and those driving large scale diversity patterns. Hence, the study of local segregation requires a better understanding of processes causing nestedness in diatom communities. Nestedness has already been found in large scale studies of diatom assemblages. Tornes and Ruhi [58] demonstrated the link between nestedness of Mediterranean river diatoms and hydrological stability. Therefore, nestedness might be related to the temporal successions described before, as a result of the accumulation of species through time in the most stable communities, especially if early successional species are still detected after species successional turnover. Soininen [59] drew a link between nestedness and dispersal processes in boreal streams. Therefore, metacommunity and spatial processes should also be explored as potential hypotheses to explain our results.

### Metacommunity perspective

The dbRDA results showed a complex but significant relationship between the environmental variables and the composition of diatom communities. This relationship was not sufficient to explain the cooccurrence patterns, and the large-scale distribution of diversity seemed to have an effect on local cooccurrences. From the different metacommunity perspectives described by Leibold et al [5], mass effect, including both environmental filters and dispersal processes, is the one that seems to match best with our results. Mass effect is a process arising due to the high rates of propagule influx, allowing species to become established in unfavorable sites [60]. Due to the extremely high number of propagules present [44], mass effect is likely to affect diatom assemblages, as well as most microbial communities. Recently, Göthe et al. [61] proposed mass effect as a process explaining the spatial structure of benthic diatom assemblages more convincingly than dispersal limitation. Here, mass effect could explain our overall aggregative pattern when compared to expectations from environmental preferences and the strong relationships between patterns of commonness of species and their local cooccurrences as well. The influx of large number of propagules from upstream has a smoothing effect on the strong segregations driven by different environmental preferences. In fact, one can easily imagine that strong mass effect from close upstream communities may have influenced colonization patterns, resulting in the aggregated and nested pattern we documented (see also [11]). Moreover, it would explain why the standard null models with fixed sums of lines and columns (NullModFF) showed the patterns closest to the real matrices, because such processes causing sampling effects are “embedded” in the models [8]. The mass effect paradigm is compatible with our results, however, further research would be needed to confirm its importance in structuring cooccurrence patterns of diatom (and other microorganism) communities. More specifically, testing and documenting the spatial structure of communities with regard to the mass effect paradigm should allow the processes causing the observed patterns to be better characterized. The use of Principal Coordinates of Neighbor Matrices [62] could help defining the scales of spatial dependence in diatom communities. In addition to standard and environmentally constrained null models, spatially constrained null models, based on kriging prediction of species’ presence might prove useful to delineate the effect of spatial processes on species cooccurrence. Finally, the analysis of the uncertainties and local residuals of the predictive models may help understanding their statistical properties and the nature of the processes causing the patterns described in our study.

### Conclusion

Although the combined use of standard and environmentally constrained null models does not provide a direct test of the processes structuring cooccurrences, it sheds light on the patterns created by these processes on a large scale with observational data. Used on diatom assemblages, this methodology shows that there are assembly rules inherent to these micro-organismal assemblages that structure cooccurrence. These assembly rules, instead of following the classical prediction of limiting similarity, tend to allow more cooccurrences between similar species and to be related with the large-scale distribution of alpha-diversity (i.e., nestedness). Temporal dynamics and mass-effect would both allow some species to become installed at the fringe of their optimal environmental conditions and explain our general aggregation patterns. The different aspects of the observed patterns and our results seem all to be consistent with strong temporal dynamics in diatom biofilms, and with the mass effect paradigm. The patterns described in this study should be considered with care when relationships between environmental data and microorganisms are constructed for bioassessment purposes.

## Supporting Information

S1 Table Diatom assemblage dataset. The dataset is described in the material and methods section.
(CSV)

S2 Table Environment dataset. The dataset is described in the material and methods section, row names correspond to those of the diatom assemblage dataset.
(CSV)

S3 Table Table of F-values for the different species guilds including species from unknown guilds. (DOC)
