# Supplementary material for: Diatom Cooccurrence Shows Less Segregation than Predicted from Niche Modeling
Source: PLoS One. 2016 Apr 29;11(4):e0154581. doi: 10.1371/journal.pone.0154581 (PMC4851409; doi:10.1371/journal.pone.0154581)
Supplement: S3 Table — (DOC) [file pone.0154581.s003.doc]

**Table 4bis. Average F-values for checkerboard units in the different models depending on species’ guilds, including unkown guilds (“Unknown”)**.

|  | NullModFF | NullModFE | LogitPP | LogitPE | RandForPP | RandForPF |
| --- | --- | --- | --- | --- | --- | --- |
| Total | 0.97 | 1.09 | 1.08 | 1.25 | 1.19 | 1.32 |
| Low-High | 0.93 | 1.04 | 1.06 | 1.19 | 1.14 | 1.26 |
| Low-Motile | 0.94 | 1.06 | 1.05 | 1.21 | 1.14 | 1.28 |
| Low-Unknown | 1.01 | 1.13 | 1.07 | 1.30 | 1.20 | 1.38 |
| High-Motile | 0.98 | 1.10 | 1.08 | 1.23 | 1.19 | 1.30 |
| High-Unknown | 0.94 | 1.04 | 1.06 | 1.24 | 1.17 | 1.29 |
| Motile-Unknown | 1.02 | 1.13 | 1.09 | 1.32 | 1.23 | 1.40 |
| Low-Low | 0.94 | 1.06 | 1.08 | 1.23 | 1.14 | 1.30 |
| High-High | 0.99 | 1.11 | 1.09 | 1.23 | 1.20 | 1.30 |
| Motile-Motile | 1.03 | 1.16 | 1.12 | 1.31 | 1.24 | 1.39 |
| Unknown-Unknown | 1.08 | 1.17 | 1.07 | 1.33 | 1.22 | 1.42 |
